# Integrated bioinformatics analysis and biological experiments to identify key immune genes in vascular dementia

**DOI:** 10.3389/fimmu.2025.1560438

**Published:** 2025-03-24

**Authors:** Yilong Zhao, Wen Xing, Weiqi Chen, Yilong Wang

**Affiliations:** ^1^ Department of Neurology, Beijing Tiantan Hospital, Capital Medical University, Beijing, China; ^2^ China National Clinical Research Center for Neurological Diseases, Beijing, China; ^3^ Department of Clinical Laboratory, Beijing Bo’ai Hospital, China Rehabilitation Research Center, Beijing, China; ^4^ School of Rehabilitation, Capital Medical University, Beijing, China; ^5^ Key Laboratory of Protein and Peptide Pharmaceuticals and Laboratory of Proteomics, Institute of Biophysics, Chinese Academy of Sciences, Beijing, China; ^6^ University of Chinese Academy of Sciences, Beijing, China; ^7^ Chinese Institute for Brain Research, Beijing, China; ^8^ National Center for Neurological Disorders, Beijing, China; ^9^ Advanced Innovation Center for Human Brain Protection, Capital Medical University, Beijing, China; ^10^ Beijing Laboratory of Oral Health, Capital Medical University, Beijing, China; ^11^ Laboratory for Clinical Medicine, Capital Medical University, Beijing, China

**Keywords:** vascular dementia, RAC1, CMTM5, bioinformatics, machine learning

## Abstract

**Objectives:**

This study aimed to identify key immune genes to provide new perspectives on the mechanisms and diagnosis of vascular dementia (VaD) based on bioinformatic methods combined with biological experiments in mice.

**Methods:**

We obtained gene expression profiles from a Gene Expression Omnibus database (GSE186798). The gene expression data were analysed using integrated bioinformatics and machine learning techniques to pinpoint potential key immune-related genes for diagnosing VaD. Moreover, the diagnostic accuracy was evaluated through receiver operating characteristic curve analysis. The microRNA, transcription factor (TF), and drug-regulating hub genes were predicted using the database. Immune cell infiltration has been studied to investigate the dysregulation of immune cells in patients with VaD. To evaluate cognitive impairment, mice with bilateral common carotid artery stenosis (BCAS) were subjected to behavioural tests 30 d after chronic cerebral hypoperfusion. The expression of hub genes in the BCAS mice was determined using a quantitative polymerase chain reaction(qPCR).

**Results:**

The results of gene set enrichment and gene set variation analyses indicated that immune-related pathways were upregulated in patients with VaD. A total of 1620 immune genes were included in the combined immune dataset, and 323 differentially expressed genes were examined using the GSE186798 dataset. Thirteen potential genes were identified using differential gene analysis. Protein-protein interaction network design and functional enrichment analysis were performed using the immune system as the main subject. To evaluate the diagnostic value, two potential core genes were selected using machine learning. Two putative hub genes, Rac family small GTPase 1(*RAC1*) and CKLF-like MARVEL transmembrane domain containing 5 (*CMTM5*) exhibit good diagnostic value. Their high confidence levels were confirmed by validating each biomarker using a different dataset. According to GeneMANIA, VaD pathophysiology is strongly associated with immune and inflammatory responses. The data were used to construct miRNA hub gene, TFs-hub gene, and drug-hub gene networks. Varying levels of immune cell dysregulation were also observed. In the animal experiments, a BCAS mouse model was employed to mimic VaD in humans, further confirmed using the Morris water maze test. The mRNA expression of *RAC1* and *CMTM5* was significantly reduced in the BCAS group, which was consistent with the results of the integrated bioinformatics analysis.

**Conclusions:**

*RAC1* and *CMTM5* are differentially expressed in the frontal lobes of BCAS mice, suggesting their potential as biomarkers for diagnosing and prognosis of VaD. These findings pave the way for exploring novel molecular mechanisms aimed at preventing or treating VaD.

## 1 Introduction

Vascular dementia (VaD) is the second leading cause of dementia after Alzheimer’s disease (AD), accounting for at least 20% of all dementia diagnoses (1). The primary manifestations of VaD include cognitive impairment, as well as behavioural and psychological symptoms (2). The persistent decline in the quality of life induced by VaD imposes significant medical and economic burdens on patients, families, and society. Therefore, an unmet need exists to investigate the molecular causes and prospective diagnostic indicators of VaD.

Bioinformatics is an interdisciplinary field that combines biology, computer science, and information technology (3). The discipline not only assists researchers in processing and analysing large-scale biological data but also provides new insights and methods for the diagnosis, treatment, and prevention of diseases (4). For instance, through bioinformatics analysis, researchers can identify genes and biomarkers associated with AD, thereby supporting personalized medicine (5). A previous study using integrative systems biology analysis demonstrated that short-chain acylcarnitines/amino acids and medium/long-chain acylcarnitines are most closely related to AD, which is mediated by adenosine triphosphate-binding cassette transporter A1 and carnitine palmitoyltransferase 1A (6). In addition, three cerebrospinal fluid (CSF) proteins, CSF sodium-/potassium-transporting ATPase subunit beta-1, serglycin, and thioredoxin-dependent peroxide reductase mitochondria, may serve as potential novel diagnostic biomarkers (7). However, VaD has not garnered significant attention.

Machine learning, an essential branch of artificial intelligence, leverages algorithms to learn from data and make predictions, and has been widely applied in data analysis across various domains (8). In bioinformatics, machine learning is employed for extracting valuable information from complex biological data, building predictive models, and identifying patterns (9). The application of machine learning has enabled bioinformatics research to process big data more efficiently, driving significant progress in areas such as disease prediction and drug discovery (10).

The objective of this study was to screen and identify key genes associated with the occurrence and progression of VaD through comprehensive bioinformatics analysis. Additionally, the analysis also aimed to investigate the pathological mechanism of VaD. Initially, we acquired the gene expression profiles of patients with VaD and healthy brain samples from a public database. Subsequently, we identified significant modules and differentially expressed immune-related genes in VaD, and screened out the key VaD genes using machine learning algorithms. Moreover, we also assessed the expression of key genes in mice with bilateral common carotid artery stenosis (BCAS) and discovered that their expression was decreased in such mice, suggesting that these genes significantly contributed to the onset and development of VaD. Thereby offering novel insights for further investigation into the pathogenesis and diagnosis of VaD.

## 2 Materials and methods

### 2.1 Microarray data source

Datasets GSE186798 (11) and GSE122063 (12) were downloaded from the Gene Expression Omnibus (GEO) database. The microarray data from GSE186798 included 10 samples each from vascular dementia-associated post-stroke dementia, post-stroke non-dementia, and healthy controls. Only fit and post-stroke dementia samples were selected from this dataset. All brain tissue samples were obtained from the frontal brain regions. The GSE122063 microarray data used as the validation set comprised brain samples from 44 healthy individuals, 36 individuals with VaD, and 56 with AD. Only gene expression data from the frontal lobe of 18 patients with VaD and 22 matched controls were retained. The GSE186798 dataset was sequenced using GPL23159, and GSE122063 using GPL16699, both of which are derived from the human body.

The ImmPort (13), GeneCards (14), and Molecular Signatures Databases (MSigDB) (15) were used to obtain 1791, 15898, and 20741 immune-related genes, respectively. Finally, 1620 immune-related genes were identified at the intersection of the three gene sets.

### 2.2 Identification of VaD-associated immune-differentially expressed genes

Differential gene analysis was performed using the R package “limma” to determine the DEGs between control and VaD samples (16). The thresholds for differential genes were set at a log2 fold change (log2FC) > 0.5 and a p-value < 0.05, indicating DEGs with increased expression. Potentially downregulated DEGs were represented by log_2_FC < −0.5 and p-value < 0.05. Volcano plots were employed to illustrate the results of differential gene expression. Differentially expressed immune genes were identified by intersecting DEGs and immune genes.

### 2.3 Gene set enrichment analysis and gene set variation analysis

GSEA was performed to identify significantly enriched functional gene sets using the R package “clusterProfiler” (17). This method determines whether a priori-defined gene set demonstrates statistically significant concordant differences between two biological states or phenotypes (18). The MSigDB contains functional annotations of the gene sets used in GSEA. Additionally, the dataset was analysed simultaneously for multiple gene sets. The gene set was considered significantly enriched if a result of a nominal P-value < 0.05 and a Q-value of < 0.25 was acquired. Gene sets meeting the abovementioned conditions were sorted from high to low according to enrichment score (ES).

GSVA is a method used to estimate the variation of gene set enrichment across samples in an expression dataset, conducted utilising the R package “GSVA” (19). The ES for gene signatures was then calculated using GSVA. Between-group differences in gene set enrichment were determined using the Limma package. Only the gene set with a P-value of < 0.05 was considered significant.

### 2.4 Function enrichment analysis

Gene Ontology (GO) annotation of the DEGs, consisting of biological process (BP), cellular component (CC), and molecular function (MF) terms, was performed using the R package “clusterProfiler”. The top three BP, CC, and MF outcomes were illustrated as Laplace plots utilising ggplot2, with a cutoff criterion of P < 0.05.

Functional enrichment of the Kyoto Encyclopedia of Genes and Genomes (KEGG) pathways for DEGs was performed using the R package “clusterProfiler”. Moreover, ggplot2 generated Laplace plots for the top three significant KEGG pathways based on P< 0.05.

### 2.5 Protein-protein interaction network

The STRING online database was utilized to determine interacting genes (20). To fully comprehend the functional interactions between proteins and select essential hub genes, the PPI network was visualised and analysed using the Cytoscape software. The MCODE algorithm was used to search for highly clustered subnetworks (21).

### 2.6 Machine learning

Two machine learning algorithms were used to investigate candidate genes for VaD diagnosis. The Least Absolute Shrinkage and Selection Operator (LASSO) is recognised as a type of penalised regression that can be used to screen variables and strengthen prediction accuracy (22). Random forest (RF), recognised as a feature selector, ranks genes based on their impact on the accuracy of the RF (23). This method forecasts continuous variables and identifies patterns with noticeable variations (24). The LASSO regression and RF analysis were carried out utilising the R packages “glmnet” (25) and “randomForest” (26). Intersecting genes identified by LASSO and RF were proposed as potential hub genes for VaD diagnosis. The positions of the identified genes were mapped to chromosomes using R packages “circlize” (27).

### 2.7 Hub genes validated by gene expression values and receiver operating characteristic analysis

To verify the expression of hub genes in the diseased and normal states, the GSE186798 and GSE122063 datasets were used as the internal and external validation sets, respectively. T-test and the “ggplot2” package of R language were used to assess and illustrate the expression of hub genes. Box plots were generated to ascertain whether the predicted VaD target genes exhibited significant differential expression in the validation set.

The “pROC” package was employed to evaluate the diagnostic predictive significance of essential genes (28). VaD was identified using the area under the curve (AUC) and 95% confidence interval (CI). An AUC >0.7 was regarded as a potential diagnostic value (29).

### 2.8 Immune infiltration analysis

CIBERSORT, a computational method utilising tissue gene expression profiles, was used to ascertain the proportion of immune cells in the samples (30). We calculated 22 immune cell types using the CIBERSORT method in the R package. A violin plot was generated to reveal striking differences in the relative compositions of immune cell populations between the VaD and control groups. Spearman’s correlation was utilized to examine the association between immune cell subtypes.

### 2.9 GeneMANIA analysis and prediction of potential microribonucleic acid, transcription factor, and drugs related to hub genes

The GeneMANIA database was used to construct networks that generated gene function hypotheses, examined gene lists, and determined gene priority through functional analysis (31). NetworkAnalyst, JASPAR, and Comparative Toxicogenomics Database (CTD) were used to predict potential miRNAs, TF, and drugs associated with hub genes. NetworkAnalyst is a comprehensive visualisation and analysis tool for gene expression data (32). JASPAR is a database of nucleotide profiles describing the binding preferences of TF, which provides predicted TF-deoxyribonucleic acid (DNA) interactions (33). The CTD is a comprehensive, publicly accessible database that offers manually curated information regarding chemical–gene/protein interactions, chemical–disease associations, and gene–disease correlations.

### 2.10 Experimental animals

The use of all animal experiments was approved by the Animal Care and Use Committee of Beijing Neurosurgical Institute. Male C57BL/6 mice aged 8–10 weeks (22–25 g) were purchased from Charles River Laboratory Animal Technology Co., Ltd. (Beijing, China). The mice were housed at the Laboratory Animal Center of the Beijing Neurosurgical Institute with free access to water and lab chow and were maintained under a 12-h light/dark cycle. The mouse model of BCAS was generated following the methods previously described (34). The mice were randomly divided into the following groups (N=7 mice per group): (1) BCAS: both common carotid arteries (CCAs) were constricted with micro-coils of an internal diameter of 0.18 mm; (2) Sham: same surgical procedure as that applied for the BCAS group except for the implantation of micro-coils.

### 2.11 Morris water maze test

Cognitive function was assessed using the MWM test on day 30 after BCAS. The MWM test was performed as previously described (35, 36). Briefly, the MWM involved a round water tank divided into four quadrants. The water temperature was maintained at 24 ± 2°C. The escape platform (10 cm in diameter) was fixed in the centre of the southwest quadrant (target quadrant) and immersed approximately 1 cm beneath the surface of the water. During the training session, mice were delicately positioned into the water maze, released facing the wall from one of the four quadrants in random order, and allowed to swim freely in search of the hidden platform. The mice were provided 60 s to discover the hidden platform, and the latency to reach the escape platform was recorded. If the mouse failed to find the platform within 60 s, it was guided to the platform and allowed to remain there on the platform 30 s. Each mouse was tested four times daily, beginning from different quadrants, with 40-min intervals between trials. Additionally, the mean was calculated as the daily score. The probe trial was conducted 24 h after the final training trial. Each mouse was allowed to swim freely in the tank and the platform was removed for 60 s. The latency to find the target quadrant, the frequency with which the mouse crossed the original platform, and the swimming velocity were automatically recorded using video tracking software (EthoVision, Noldus, Netherlands).

### 2.12 Quantitative real-time polymerase chain reaction

Brain tissue was rapidly removed from the frontal lobe, and the messenger RNA (mRNA) levels of RAC1 and CMTM5 were measured using qRT-PCR. Total RNA was isolated using the TRIzol reagent, and complementary DNA synthesis was conducted using a Revert Aid First Strand complementary DNA Synthesis Kit (Yeasen) in accordance with the manufacturer’s guidelines. qRT-PCR was performed using SYBR Green Real-Time PCR Master Mix (Yeasen) on a StepOne Plus Real-Time PCR System (Applied Biosystems). Glyceraldehyde-3-phosphate dehydrogenase was used as a reference gene. All quantitative PCR was performed in triplicate using seven independent purified RNA samples. The primer sequences are listed in **Table 1**.

**Table 1:** Primers used for qRT-PCR.

Gene	Primer sequences (5’-3’)	
RAC1	F: GGACACCATTGAGAAGCTGAAGG	R: GTCTTGAGTCCTCGCTGTGTGA
CMTM5	F: TTCGGAGTGGACAAGACCTTCC	R: CCAGTGTGATGAGGAACTCTAGC
GAPDH	F: CATCACTGCCACCCAGAAGACTG	R: ATGCCAGTGAGCTTCCCGTTCAG

### 2.13 Data expression and statistical analysis

Statistical analyses were performed using GraphPad Prism software (version 7.00; GraphPad Software Inc., USA). All data were presented as the mean ± standard error of the mean. Comparisons between groups were statistically evaluated using Student’s t-test. Statistical significance was set at P < 0.05.

## 3 Results

### 3.1 Data processing and identification of VaD-related pathways

**Figure 1:**
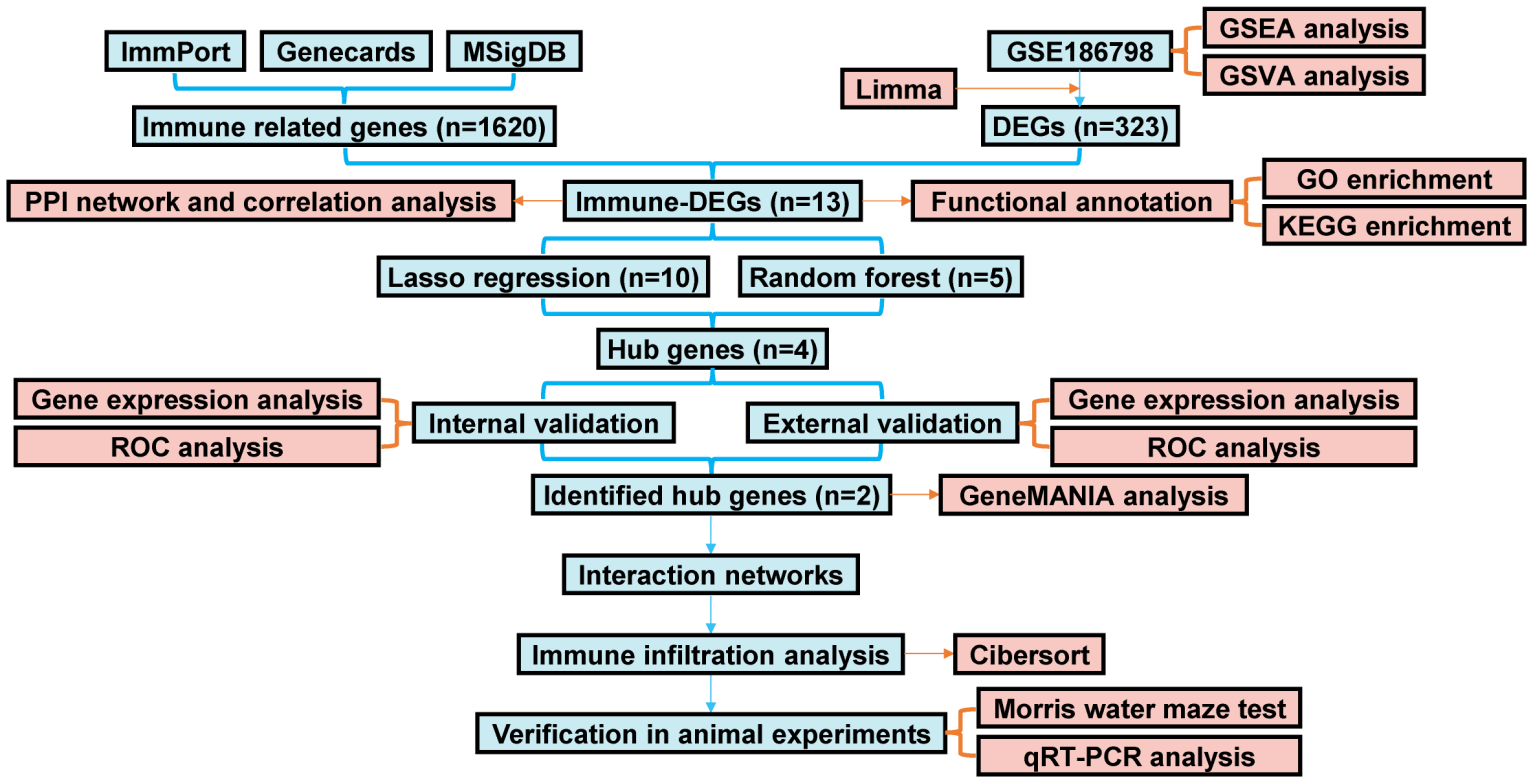
The flow chart of the study.

**Figure 2:**
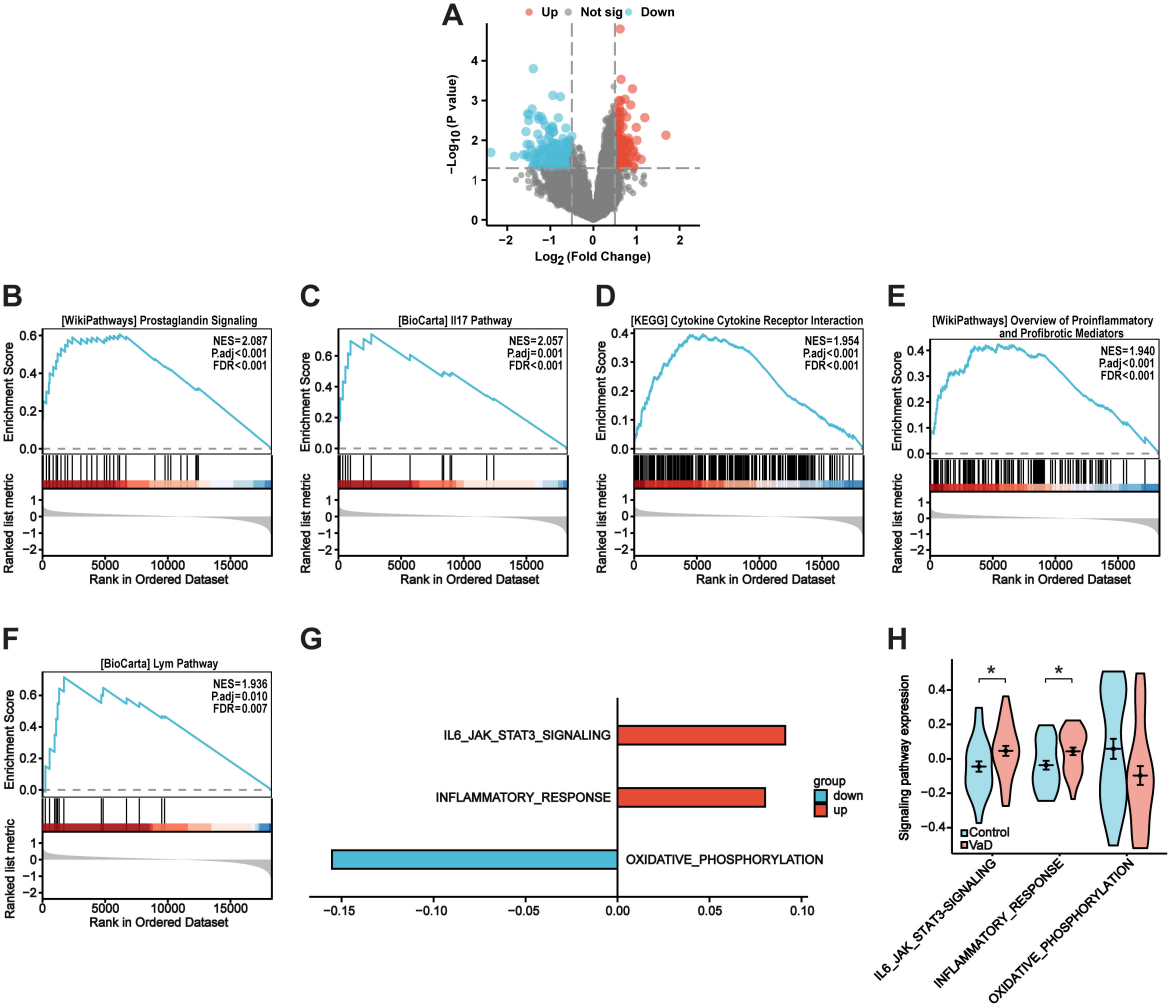
Identification of differentially expressed genes and VaD-Related pathways. **(A)** VaD-related differentially expressed genes (DEGs) volcano plot with log_2_FoldChange in the horizontal coordinate and -log_10_(P-value) in the vertical coordinate. Red nodes indicate upregulated DEGs, blue nodes indicate downregulated DEGs, and gray nodes indicate genes that are not significantly differentially expressed. **(B-F)** GSEA analysis showing the top five pathways associated with VaD. **(G)** GSVA analysis illustrating VaD-related pathways. **(H)** Violin plot showing the expression level of VaD-related pathways. Blue for control samples, red for VaD samples. Data represent the mean ± SEM. *P<0.05.

**Figure 1** depicts the flow chart for the bioinformatics analysis in this study. Information sets were obtained from the GEO database (GSE186798), including brain samples from 10 patients with VaD and 10 controls. In the comparison of VaD and control samples, 323 DEGs were identified, of which 138 were upregulated and 185 were downregulated. **Figure 2A** illustrates a volcano plot of the DEGs. Furthermore, GSEA revealed distinct upregulated gene sets linked to VaD, and the top five pathways were selected. We identified that the immune-related pathways were significantly activated in the VaD group, including “prostaglandin signalling”, “interleukin (IL)-17 pathway”, “cytokine-cytokine receptor interaction”, “overview of inflammatory and profibrotic mediators”, and “lym pathway” (**Figures 2B–F**). The GSVA revealed similar results. Moreover, “IL6-JAK-STAT signalling and “inflammatory responses” were highly activated in the VaD group, whereas oxidative phosphorylation was hyperactivated in the control group (**Figures 2G, H**).

### 3.2 Screening of immune-associated DEGs, functional enrichment analysis and PPI construction

**Figure 3:**
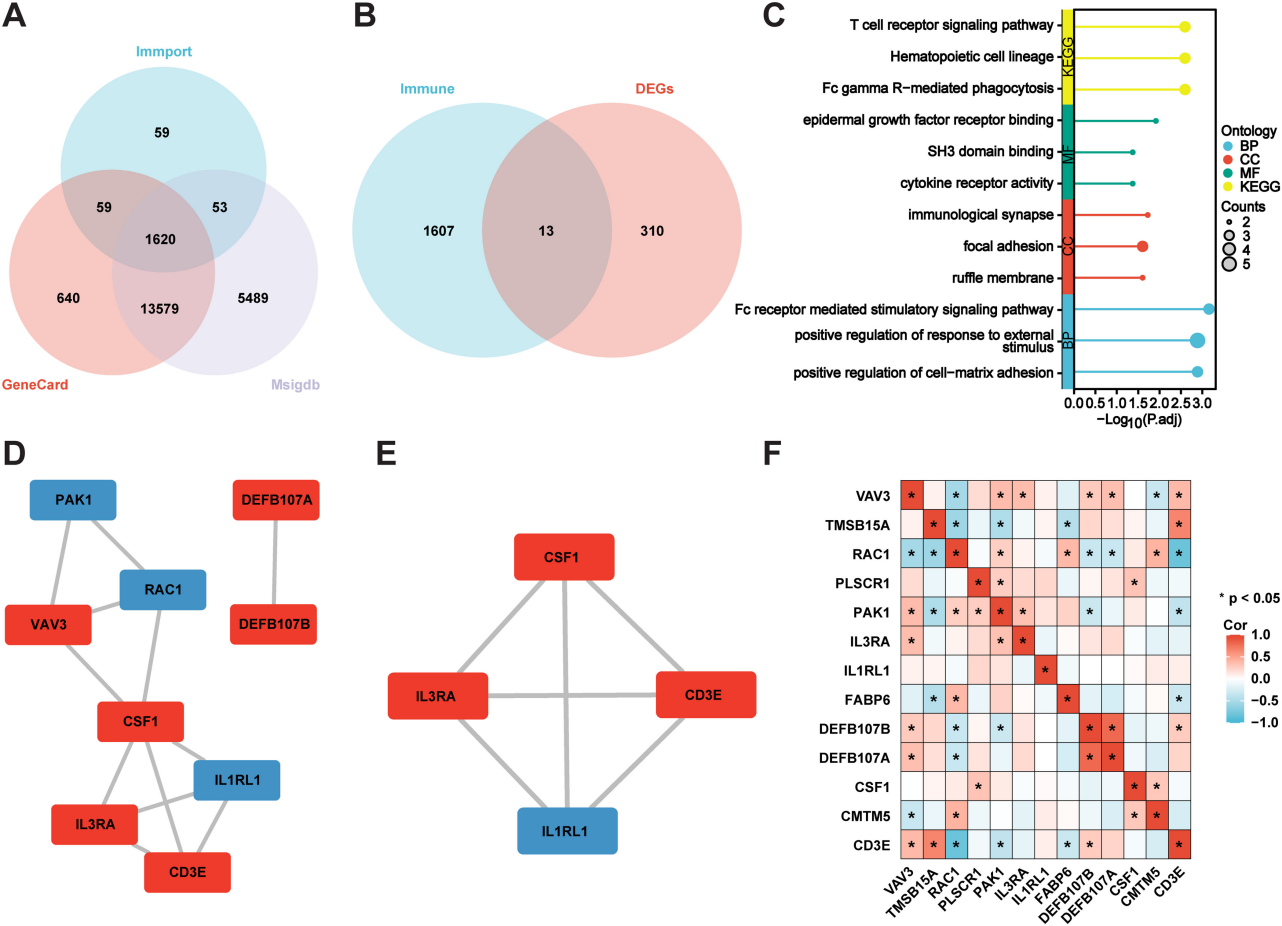
Identification of immune-associated DEGs, and enrichment and correlation analysis. **(A)** Immune genes Venn’s diagram. **(B)** Immune genes and DEGs Venn’s diagram. **(C)** GO and KEGG analysis of the genes immune-associated DEGs. **(D, E)** PPI network of the immune-associated DEGs, and the MCODE plug-in is used to depict the most important module. Blue for downregulated genes, red for upregulated genes. **(F)** Correlation Analysis of the immune-associated DEGs. *P<0.05.

The immune-related genes of the three immune datasets interacted with the DEGs to identify 13 immune-related DEGs (**Figures 3A, B**). According to KEGG analysis, “T cell receptor signalling pathway”, “Hematopoietic cell lineage” and “FC gamma R-mediated phagocytosis” were the three conditions where common genes (CGs) were most highly enriched. GO analysis indicated that CGs were predominantly enhanced in BP terms, including “FC receptor-mediated stimulatory signalling pathway”, “positive regulation of response to external stimulus”, and “positive regulation of cell-matrix adhesion”. Concerning CC metaphysics, the CGs primarily settled within the “immunological synapse”, “focal adhesion”, and “ruffle membrane”. MF analysis demonstrated that “epidermal growth factor receptor binding”, “SH3 domain binding”, and “cytokine receptor activity” were crucial elements in the metric system (**Figure 3C**). Enrichment analysis revealed that VaD was primarily associated with inflammatory responses, which strongly correlated with the pathological progression of the disease.

We constructed a PPI network to identify node genes and searched for densely connected subnetworks. As depicted in Cytoscape, the PPI network of the immune DEGs contained 12 paired interactions and involved nine genes. Node genes were displayed using the MCODE plug-in (**Figures 3D, E**). Additionally, we conducted a correlation analysis of the immune DEGs. DEFB107A and DEFB107B demonstrated a notable positive correlation, while RAC1 and CD3E exhibited a significant negative correlation (**Figure 3F**).

### 3.3 Identifying potential hub genes using machine learning

**Figure 4:**
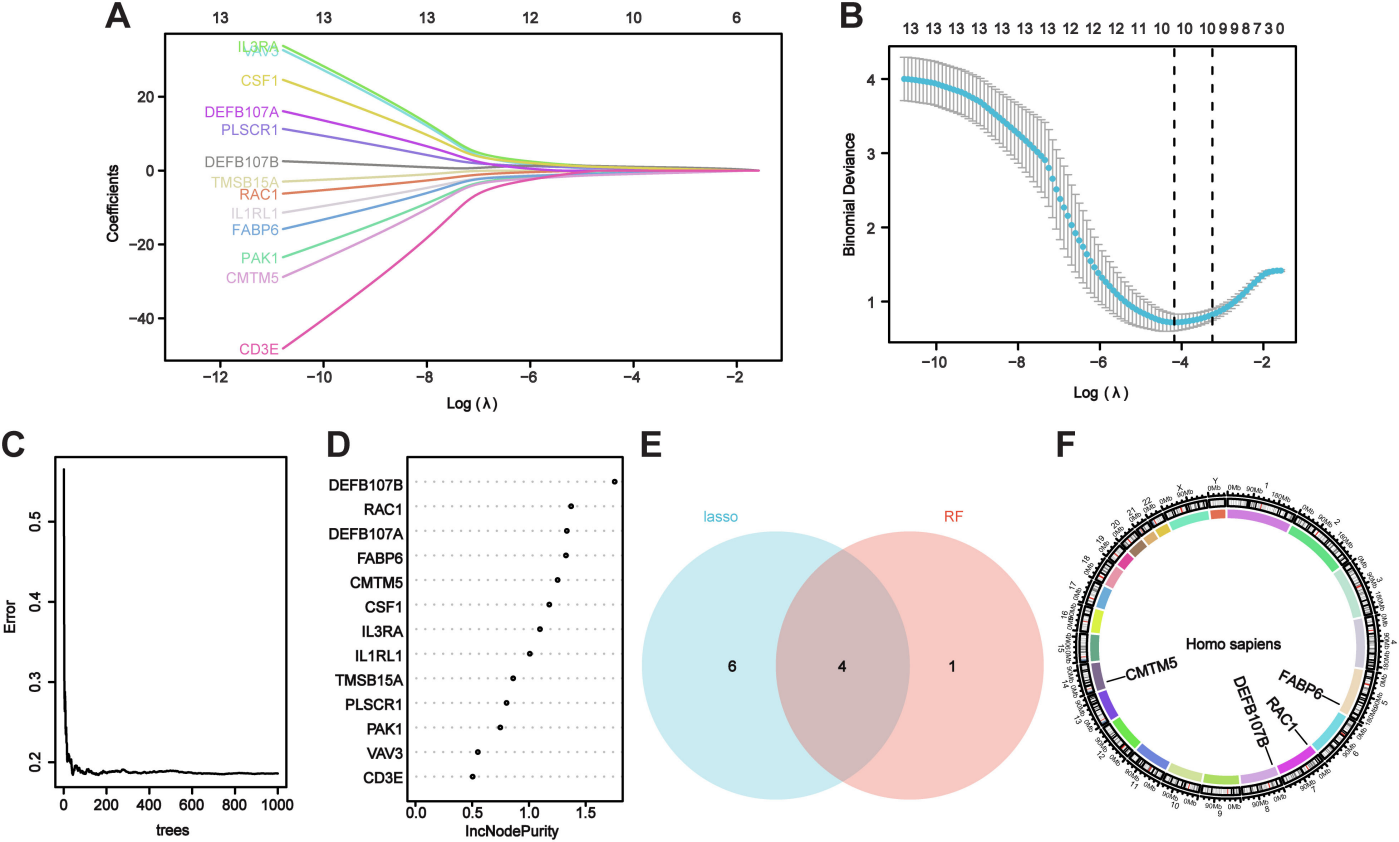
Evaluating hub genes for VaD using machine learning. **(A, B)** The Lasso model’s biomarker screening. **(C, D)** The random forest algorithmic program displays the error, and variable importance plot. **(E)** The Venn figure illustrates two methods used to identify four candidate diagnostic genes. **(F)** Position of candidate diagnostic genes on chromosomes.

Candidate genes were screened utilizing LASSO regression and RF machine learning methodologies for diagnostic value assessment. **Figures 4A, B** demonstrate that the LASSO regression algorithm identified 10 probable candidate biomarkers. **Figures 4C, D** demonstrate that the RF algorithmic program systematically arranged the sequences to facilitate the estimation of the relative importance of each gene. When the Venn diagram illustrating the overlap of the 10 most essential genes identified by LASSO and the five probable candidate genes from RF was presented (**Figure 4E**), four hub genes (*RAC1, FABP6, DEFB107B*, and *CMTM5*) were identified for final confirmation. **Figure 4F** displays the positions of hub genes on the chromosomes.

### 3.4 Validation of the hub genes

**Figure 5:**
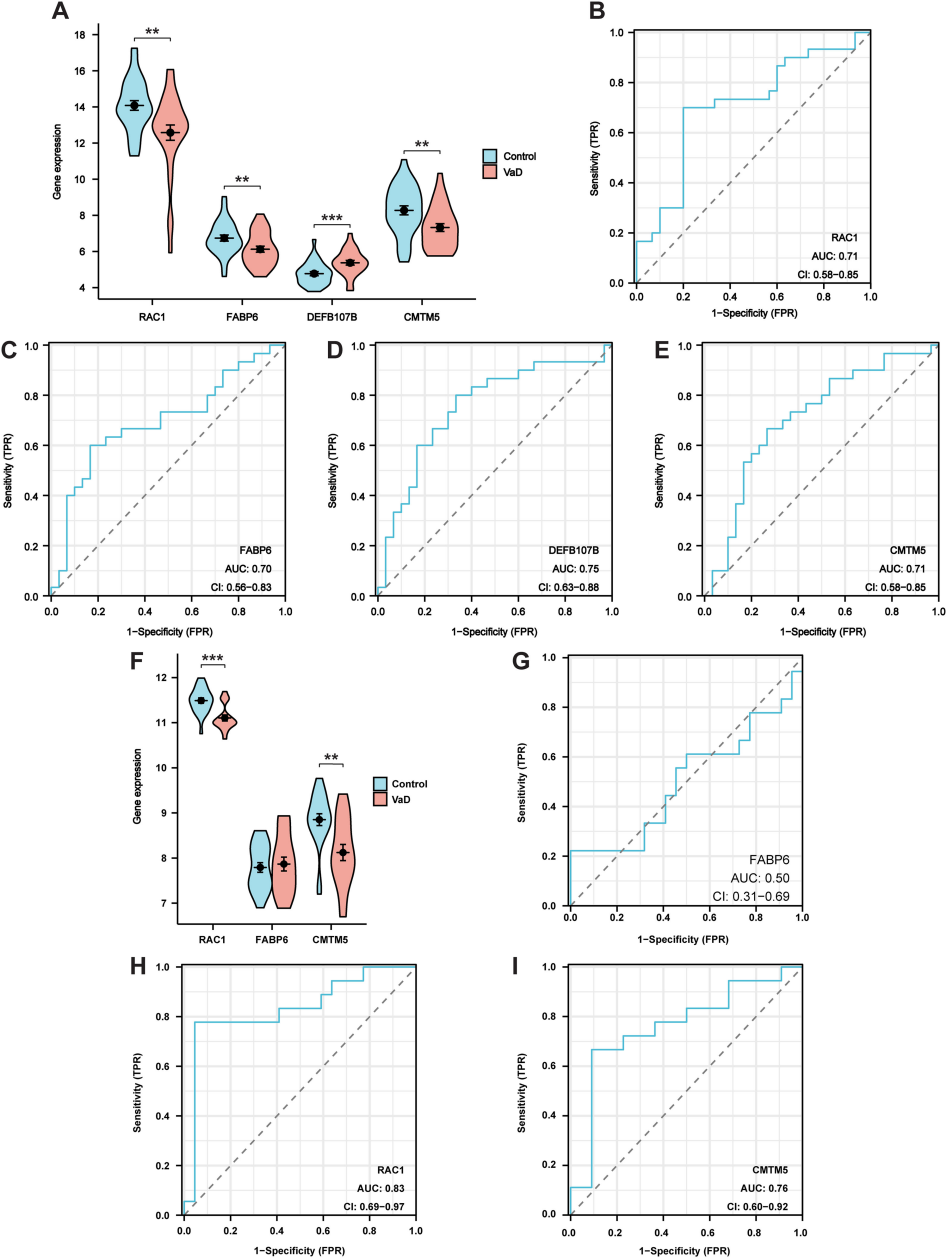
Validation of the hub genes. **(A)** Violin plot showing the expression of target genes in internal validation set. **(B-E)** ROC curves for RAC1, FABP6, DEFB107B, and CMTM5 in internal validation set. **(F)** Violin plot showing the expression of RAC1, FABP6, and CMTM5 in external validation set. **(G-I)** ROC curves for RAC1, FABP6, and CMTM5 in external validation set. Data represent the mean ± SEM. **P<0.01; ***P<0.001.

The GSE186798 dataset was used as an internal validation set to verify the reliability of hub genes. Violin plots were employed to determine the expression levels of screened hub genes. The results demonstrated that the expression of RAC1, FABP6, DEFB107B, and CMTM5 significantly differed in the VaD group compared to that in the control group (**Figure 5A**). Additionally, RAC1 (AUC 0.71, CI 0.58–0.85), FABP6 (AUC 0.70, CI 0.56–0.83), DEFB107B (AUC 0.75, CI 0.63–0.88), and CMTM5 (AUC 0.71, CI 0.58–0.85) had a potential diagnostic value for VaD based on the plotting of ROC curves (**Figures 5B–E**).

As DEFB107B was not tested, RAC1, FABP6, and CMTM5 were validated using an external validation set (GSE122063 dataset). Our results demonstrated that *RAC1* and *CMTM5* were significantly decreased in the VaD group compared to those in the control group (**Figure 5F**). Furthermore, ROC analysis revealed that these two hub genes, *RAC1* (AUC 0.83, CI 0.69–0.97), and *CMTM5* (AUC 0.76, CI 0.60–0.92) had a good diagnostic value for VaD. Nevertheless, the diagnostic significance of FABP6 (AUC 0.50, CI 0.31–0.69) was poor, with AUC values falling below 0.70 (**Figures 5G–I**). Therefore, hub genes *RAC1* and *CMTM5* were used in subsequent experiments.

### 3.5 GeneMANIA, and immune cell infiltration analysis

**Figure 6:**
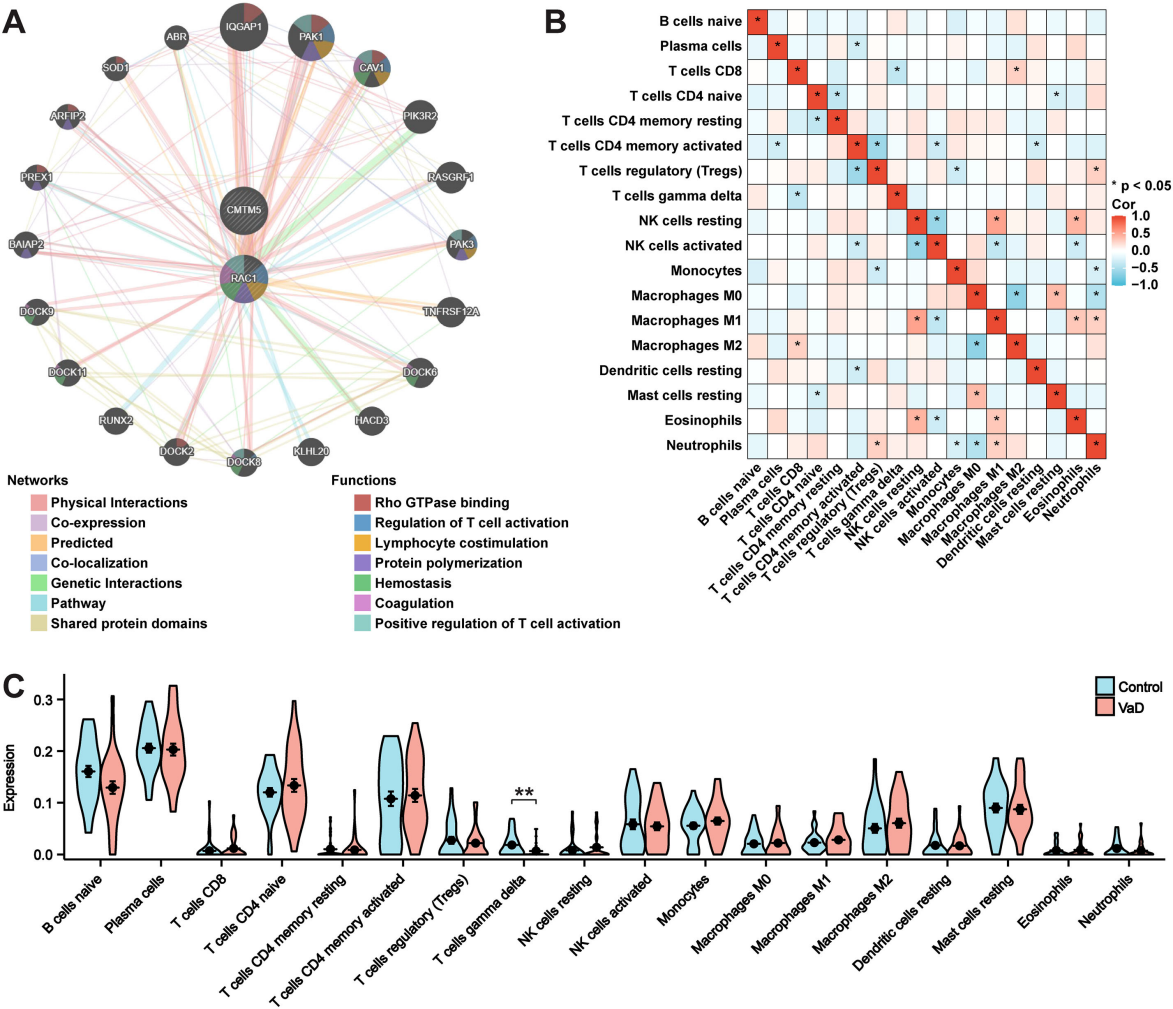
GeneMANIA, and immune cell infiltration analysis. **(A)** PPI network construction using GeneMANIA analysis. **(B)** Correlation of the cell types of 18 immune cells. **(C)** 18 categories of immune cells’ proportions in the VaD and control groups. Data represent the mean ± SEM. **P<0.01.

A functional network established using GeneMANIA indicated that these pathways were closely linked to immune and inflammatory responses (**Figure 6A**). As memory B cells, follicular helper T cells, activated dendritic cells, and activated mast cells were not expressed in the sample, they were excluded from the subsequent phase of the analysis. Macrophages M1 and resting natural killer cells exhibited a notable positive correlation. Conversely, macrophages M0 and macrophages M2 cells had a significant negative correlation. Spearman’s test was used to assess significant differences in immune cell infiltration between patients with VaD and the control group (**Figure 6B**). In addition, we evaluated the proportions of 18 immune cell subtypes between the groups. The findings revealed a strikingly different immune cell presence in the VaD group compared to that in the control group. Gamma-delta T cells were significantly reduced in patients with VaD compared to those in the controls, indicating the necessity of increasing this type of cell within the VaD immune microenvironment (**Figure 6C**).

### 3.6 Establishment of regulatory network for miRNAs, TFs, and drugs-hub genes

**Figure 7:**
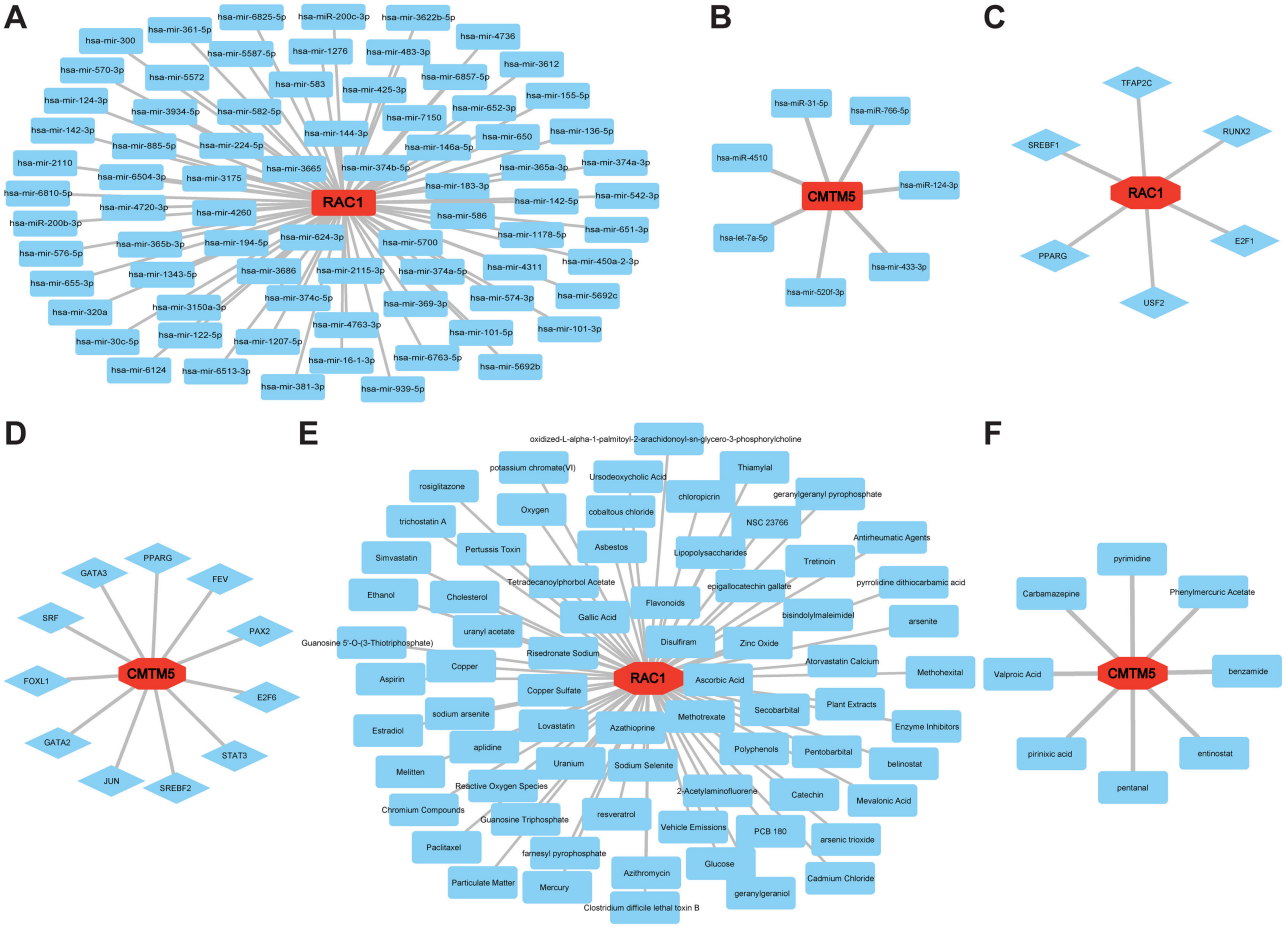
Establishment of regulatory network for MiRNAs−Hub Genes, TFs-Hub Genes, and Drugs-Hub Genes. **(A, B)** MiRNA regulatory networks for RAC1 and CMTM5. **(C, D)** TFs regulatory networks for RAC1 and CMTM5. **(E, F)** Predicted drug regulatory networks for RAC1 and CMTM5.

Furthermore, miRNAs perform various functions in the regulation of gene expression. Hub gene and miRNA regulatory networks were constructed using Cytoscape to predict miRNAs targeting hub genes based on the NetworkAnalyst database. In **Figures 7A, B**, the two hub genes and their associated regulatory miRNA molecules are displayed. Similarly, we predicted the TFs of the target genes using the JASPAR database, which could be harnessed as innovative regulators of VaD pathogenesis (**Figures 7C, D**). Moreover, the CTD was used to conduct our final drug discovery search to lay the foundation for VaD treatment (**Figures 7E, F**).

### 3.7 Vascular dementia in BCAS mice and qRT-PCR validation of data

**Figure 8:**
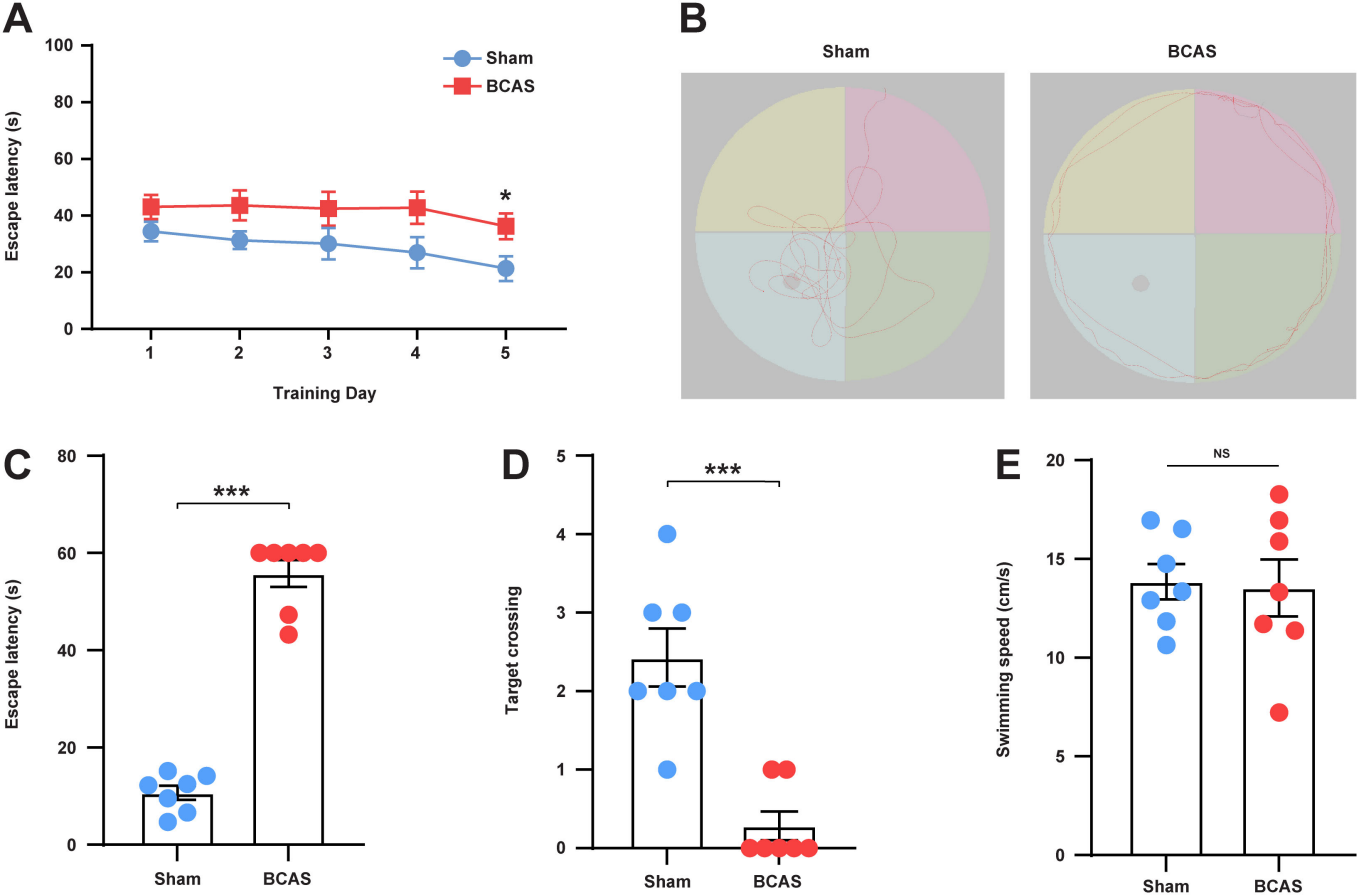
Mice subjected to CCH exhibited impaired cognitive function. **(A)** Average escape latency for each group during the training phase. **(B)** Representative swimming path of each group during the testing phase. **(C)** Average escape latency for each group during the testing phase. **(D)** Number of times crossing the platform for each group. **(E)** Average swimming speed for each group. N = 7. Data represent the mean ± SEM. NS, nonsignificant; ***P<0.001. BCAS, both common carotid arteries (CCAs) were constricted with microcoils of an internal diameter; Sham, same surgical procedure as BCAS group except for the implantation of microcoils.

To investigate whether the BCAS model induces cognitive impairment in mice, we conducted the MWM test. On day 5 of the training phase, the BCAS group exhibited a longer escape latency to the platform in comparison to that observed in the sham group (**Figure 8A**). Representative images of the traces from the spatial probe assessment are displayed in **Figure 8B**. Compared with the sham group, escape latency (**Figure 8C**) was significantly higher and the frequency of times crossing the target (**Figure 8D**) was significantly lower in the BCAS group, indicating that cognitive dysfunction was triggered by chronic cerebral hypoperfusion (CCH). Furthermore, no significant difference was observed in swimming velocity between the two groups (**Figure 8E**), suggesting that the MWM test performance was not affected by variations in swimming, motor ability, or motivational deficits.

**Figure 9:**
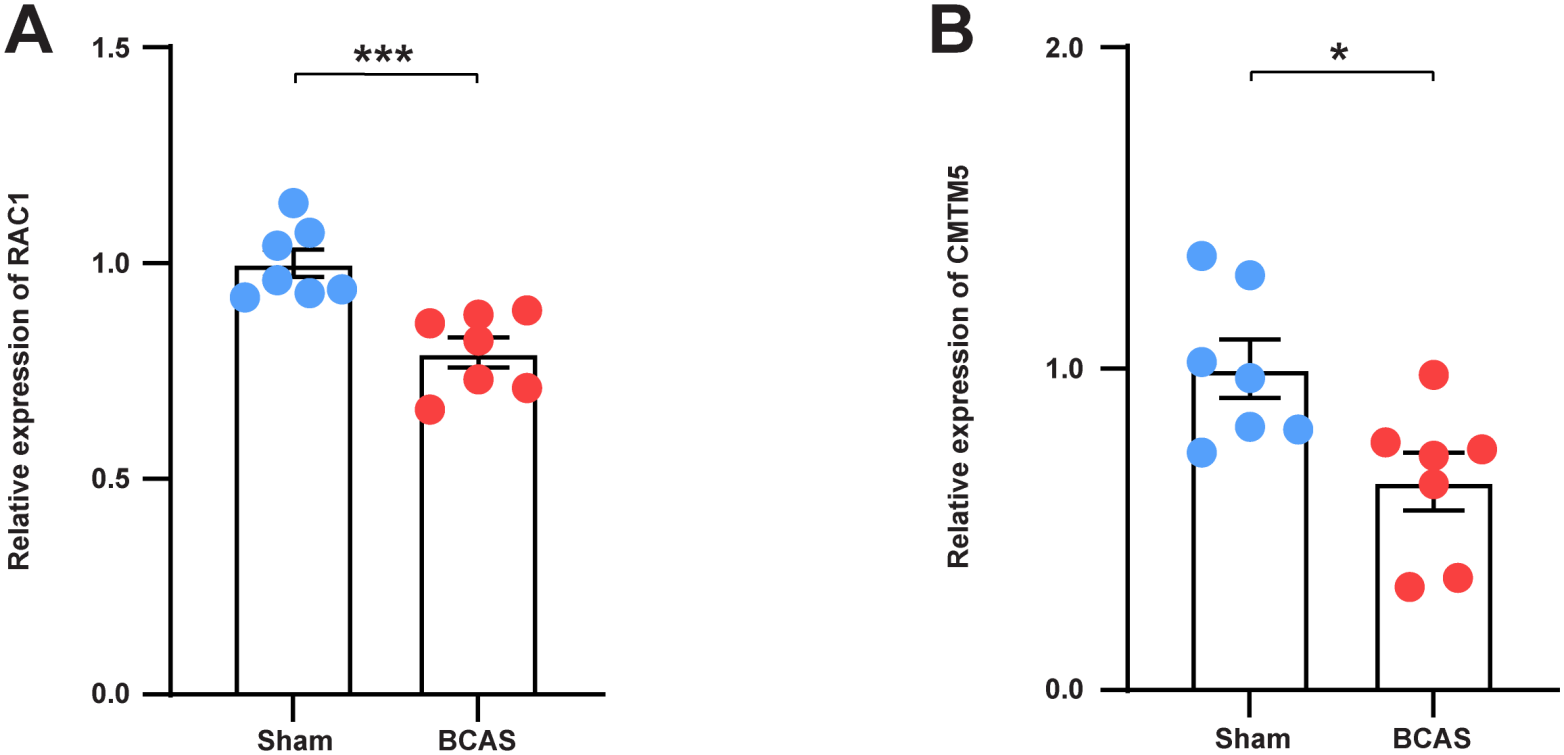
Validation of the expression of hub genes in BCAS Mice. **(A)** The mRNA levels of RAC1 in the frontal lobe of each group. **(B)** The mRNA levels of CMTM5 in the frontal lobe of each group. N = 7. Data represent the mean ± SEM. *P<0.05; ***P<0.001. BCAS, both common carotid arteries (CCAs) were constricted with microcoils of an internal diameter; Sham, same surgical procedure as BCAS group except for the implantation of microcoils.

To verify the bioinformatic findings, qRT-PCR was performed. The results demonstrated that the mRNA expression levels of RAC1 and CMTM5 were significantly lower in the VaD group than in the control group. This indicates that the data mining outcomes were reliable and have considerable research significance (**Figures 9A, B**).

## 4 Discussion

The immune response is a protective process against external stimuli or internal injuries involving various immune cells and cytokines (37). Studies have demonstrated that the immune response of the nervous system plays a crucial role in VaD (38). CCH, the leading cause of VaD (39, 40), induces endothelial dysfunction, increases blood-brain barrier permeability, and facilitates the leakage of plasma proteins into the brain, culminating in robust immune and inflammatory responses (41). Animal research on VaD induced by CCH has validated that microglia become activated following the onset of cerebral hypoperfusion, ultimately resulting in white matter damage and VaD (42, 43). Consistently, the GSEA and GSVA results of our study revealed that immune-related pathways were highly activated in the VaD group, indicating that the immune response plays an important role in the development of VaD.

The involvement of multiple molecular pathways in neuroimmune regulation gives rise to several potential biomarkers (44, 45). However, investigations of these potential biomarkers in the context of VaD have yielded inconsistent results. This inconsistency makes the application of inflammatory biomarkers in VaD problematic. To resolve this issue, we incorporated integrated bioinformatic analysis and machine learning to identify two hub genes (*RAC1* and *CMTM5*) with diagnostic potential. These results were validated using *in vivo* experiments.

RAC1, a member of the Rho GTPase family, was ubiquitously expressed (46). The activity of RAC1 within cells is regulated by its binding state with GTP (guanosine triphosphate) and GDP (guanosine diphosphate). Specifically, RAC1 is active when bound to GTP and inactive when bound to GDP (47). When activated, RAC1 facilitates the allosteric regulation of p21-activated kinase, thereby promoting its phosphorylation and subsequent activation (48). Additionally, RAC1 functions include the regulation of various physiological processes such as cytoskeletal reorganisation, cell migration, and cell adhesion (49). A previous study demonstrated that neuronal RAC1 significantly enhanced cognitive recovery after stroke by activating various signalling pathways, including promoting axonal plasticity and reducing the astrocytic barrier (50). Another study showed that RAC1 promoted axonal regeneration and alleviated cognitive dysfunction by regulating glial fibrillary acidic protein signalling (51). RAC1 is involved in NADPH oxidase activation, leading to ROS production, which influences immune signalling and antioxidant regulation (52). Our results indicated that the expression of RAC1 was reduced in VaD, which is consistent with these results.

CMTM5 is a novel tumour suppressor belonging to the CMTM family that contains a MARVEL domain in its structure and was initially reported by the Human Disease Gene Research Center of Beijing University in 2003 (53). CMTM5 played a potentially crucial role in transmembrane signalling and inhibiting tumour cell proliferation, adhesion and migration through regulating EGFR and PI3K/AKT Pathway (54, 55). However, the role of CMTM5 in VaD is not well established. To the best of our knowledge, this is the first study to demonstrate that CMTM5 expression is downregulated in the brain tissue of mice with VaD. Furthermore, we predicted miRNAs, TFs, and drugs linked to CMTM5 using internet databases, which provided clues for the diagnosis and treatment of VaD.

Our study has certain limitations. Firstly, owing to the differences in analytical thinking and approaches, our investigation may be a secondary mining of a previously released dataset, yielding divergent findings. The two hub genes screened in this study have not been experimentally validated in human brain tissue. Secondly, our analysis was based on a limited sample size, necessitating additional evidence to confirm our hypotheses. Further research with large sample size is required to ascertain the diagnostic accuracy of hub genes for VaD, to elucidate the connection between hub gene levels and future treatment strategies, and to identify the immune-related pathological mechanisms underlying VaD. Thirdly, this study utilised only the BCAS animal model for VaD, and further experiments are necessary to validate these findings in other models of VaD.

In conclusion, we identified two hub genes (*RAC1* and *CMTM5*) associated with VaD, reinforcing the potential of specific hub genes as biomarkers. These findings pave the way for future research into accurate diagnosis and enhanced understanding of VaD pathogenesis.

## Data Availability

The original contributions presented in the study are included in the article/**Supplementary Material**. Further inquiries can be directed to the corresponding authors.

## References

[1] Hosoki S Hansra GK Jayasena T Poljak A Mather KA Catts VS. Molecular biomarkers for vascular cognitive impairment and dementia. Nat Rev Neurology. (2023) 19:737–53. doi: 10.1038/s41582-023-00884-1 37957261

[2] van der Flier WM Skoog I Schneider JA Pantoni L Mok V Chen CLH. Vascular cognitive impairment. Nat Rev Dis primers. (2018) 4:18003. doi: 10.1038/nrdp.2018.3 29446769

[3] Gauthier J Vincent AT Charette SJ Derome N. A brief history of bioinformatics. Brief Bioinform. (2019) 20:1981–96. doi: 10.1093/bib/bby063 30084940

[4] Zech M Winkelmann J. Next-generation sequencing and bioinformatics in rare movement disorders. Nat Rev Neurology. (2024) 20:114–26. doi: 10.1038/s41582-023-00909-9 38172289

[5] He S Dou L Li X Zhang Y. Review of bioinformatics in azheimer's disease research. Comput Biol Med. (2022) 143:105269. doi: 10.1016/j.compbiomed.2022.105269 35158118

[6] Horgusluoglu E Neff R Song WM Wang M Wang Q Arnold M. Integrative metabolomics-genomics approach reveals key metabolic pathways and regulators of Alzheimer's disease. Alzheimer's dementia: J Alzheimer's Assoc. (2022) 18:1260–78. doi: 10.1002/alz.12468 PMC9085975 34757660

[7] Liu P Li L He F Meng F Liu X Su Y. Identification of candidate biomarkers of alzheimer's disease via multiplex cerebrospinal fluid and serum proteomics. Int J Mol Sci. (2023) 24:14225. doi: 10.3390/ijms241814225 37762527 PMC10532410

[8] Goecks J Jalili V Heiser LM Gray JW. How machine learning will transform biomedicine. Cell. (2020) 181:92–101. doi: 10.1016/j.cell.2020.03.022 32243801 PMC7141410

[9] Krittanawong C Johnson KW Rosenson RS Wang Z Aydar M Baber U. Deep learning for cardiovascular medicine: a practical primer. Eur Heart J. (2019) 40:2058–73. doi: 10.1093/eurheartj/ehz056 PMC6600129 30815669

[10] Greener JG Kandathil SM Moffat L Jones DT. A guide to machine learning for biologists. Nat Rev Mol Cell Biol. (2022) 23:40–55. doi: 10.1038/s41580-021-00407-0 34518686

[11] Waller R Hase Y Simpson JE Heath PR Wyles M Kalaria RN. Transcriptomic profiling reveals discrete poststroke dementia neuronal and gliovascular signatures. Trans stroke Res. (2023) 14:383–96. doi: 10.1007/s12975-022-01038-z PMC10160172 35639336

[12] McKay EC Beck JS Khoo SK Dykema KJ Cottingham SL Winn ME. Peri-infarct upregulation of the oxytocin receptor in vascular dementia. J neuropathology Exp neurology. (2019) 78:436–52. doi: 10.1093/jnen/nlz023 PMC6467199 30990880

[13] Bhattacharya S Dunn P Thomas CG Smith B Schaefer H Chen J. ImmPort, toward repurposing of open access immunological assay data for translational and clinical research. Sci Data. (2018) 5:180015. doi: 10.1038/sdata.2018.15 29485622 PMC5827693

[14] Stelzer G Rosen N Plaschkes I Zimmerman S Twik M Fishilevich S. The geneCards suite: from gene data mining to disease genome sequence analyses. Curr Protoc Bioinf. (2016) 54:1.30.1–1.30.33. doi: 10.1002/cpbi.5 27322403

[15] Subramanian A Tamayo P Mootha VK Mukherjee S Ebert BL Gillette MA. Gene set enrichment analysis: a knowledge-based approach for interpreting genome-wide expression profiles. Proc Natl Acad Sci United States America. (2005) 102:15545–50. doi: 10.1073/pnas.0506580102 PMC1239896 16199517

[16] Ritchie ME Phipson B Wu D Hu Y Law CW Shi W. limma powers differential expression analyses for RNA-sequencing and microarray studies. Nucleic Acids Res. (2015) 43:e47. doi: 10.1093/nar/gkv007 25605792 PMC4402510

[17] Martinez-Zamudio RI Roux PF de Freitas J Robinson L Dore G Sun B. AP-1 imprints a reversible transcriptional programme of senescent cells. Nat Cell Biol. (2020) 22:842–55. doi: 10.1038/s41556-020-0529-5 PMC7899185 32514071

[18] Shelton RC Claiborne J Sidoryk-Wegrzynowicz M Reddy R Aschner M Lewis DA. Altered expression of genes involved in inflammation and apoptosis in frontal cortex in major depression. Mol Psychiatry. (2011) 16:751–62. doi: 10.1038/mp.2010.52 PMC2928407 20479761

[19] Li H Liu P Zhang B Yuan Z Guo M Zou X. Acute ischemia induces spatially and transcriptionally distinct microglial subclusters. Genome Med. (2023) 15:109. doi: 10.1186/s13073-023-01257-5 38082331 PMC10712107

[20] Huang S. Efficient analysis of toxicity and mechanisms of environmental pollutants with network toxicology and molecular docking strategy: Acetyl tributyl citrate as an example. Sci Total Environ. (2023) 905:167904. doi: 10.1016/j.scitotenv.2023.167904 37858827

[21] Fang HT El Farran CA Xing QR Zhang LF Li H Lim B. Global H3.3 dynamic deposition defines its bimodal role in cell fate transition. Nat Commun. (2018) 9:1537. doi: 10.1038/s41467-018-03904-7 29670118 PMC5906632

[22] Collaborators C-EM. Estimating excess mortality due to the COVID-19 pandemic: a systematic analysis of COVID-19-related mortality, 2020-21. Lancet (London England). (2022) 399:1513–36. doi: 10.1016/S0140-6736(21)02796-3 PMC8912932 35279232

[23] Kwon J Kang J Jo A Seo K An D Baykan MY. Single-cell mapping of combinatorial target antigens for CAR switches using logic gates. Nat Biotechnol. (2023) 41:1593–605. doi: 10.1038/s41587-023-01686-y 36797491

[24] Ellis K Kerr J Godbole S Lanckriet G Wing D Marshall S. A random forest classifier for the prediction of energy expenditure and type of physical activity from wrist and hip accelerometers. Physiol Meas. (2014) 35:2191–203. doi: 10.1088/0967-3334/35/11/2191 PMC4374571 25340969

[25] Li Y Lu F Yin Y. Applying logistic LASSO regression for the diagnosis of atypical Crohn's disease. Sci Rep. (2022) 12:11340. doi: 10.1038/s41598-022-15609-5 35790774 PMC9256608

[26] Wang Y Zhuang H Jiang XH Zou RH Wang HY Fan ZN. Unveiling the key genes, environmental toxins, and drug exposures in modulating the severity of ulcerative colitis: a comprehensive analysis. Front Immunol. (2023) 14:1162458. doi: 10.3389/fimmu.2023.1162458 37539055 PMC10394652

[27] Kreye J Reincke SM Kornau HC Sanchez-Sendin E Corman VM Liu H. A therapeutic non-self-reactive SARS-coV-2 antibody protects from lung pathology in a COVID-19 hamster model. Cell. (2020) 183:1058–1069 e19. doi: 10.1016/j.cell.2020.09.049 33058755 PMC7510528

[28] Richards SM Guo F Zou H Nigsch F Baiges A Pachori A. Non-invasive candidate protein signature predicts hepatic venous pressure gradient reduction in cirrhotic patients after sustained virologic response. Liver international: Off J Int Assoc Study Liver. (2023) 43:1984–94. doi: 10.1111/liv.15657 37443448

[29] Zheng W Yang C Qiu L Feng X Sun K Deng H. Transcriptional information underlying the generation of CSCs and the construction of a nine-mRNA signature to improve prognosis prediction in colorectal cancer. Cancer Biol Ther. (2020) 21:688–97. doi: 10.1080/15384047.2020.1762419 PMC7515529 32453965

[30] Lukovic J Pintilie M Han K Fyles AW Bruce JP Quevedo R. An immune gene expression risk score for distant metastases after radiotherapy for cervical cancer. Clin Cancer Res. (2024) 30:1200–7. doi: 10.1158/1078-0432.CCR-23-2085 38180733

[31] Jin D Tu X Xu W Zheng H Zeng J Bi P. Identification and validation of diagnostic markers related to immunogenic cell death and infiltration of immune cells in diabetic nephropathy. Int Immunopharmacol. (2024) 143:113236. doi: 10.1016/j.intimp.2024.113236 39378654

[32] Zhou G Soufan O Ewald J Hancock REW Basu N Xia J. NetworkAnalyst 3.0: a visual analytics platform for comprehensive gene expression profiling and meta-analysis. Nucleic Acids Res. (2019) 47:W234–41. doi: 10.1093/nar/gkz240 PMC6602507 30931480

[33] Krasnov GS Dmitriev AA Melnikova NV Zaretsky AR Nasedkina TV Zasedatelev AS. CrossHub: a tool for multi-way analysis of The Cancer Genome Atlas (TCGA) in the context of gene expression regulation mechanisms. Nucleic Acids Res. (2016) 44:e62. doi: 10.1093/nar/gkv1478 26773058 PMC4838350

[34] Du Y He J Xu Y Wu X Cheng H Yu J. SIRT6 prevent chronic cerebral hypoperfusion induced cognitive impairment by remodeling mitochondrial dynamics in a STAT5-PGAM5-Drp1 dependent manner. J Transl Med. (2024) 22:788. doi: 10.1186/s12967-024-05566-0 39183280 PMC11346289

[35] Pao PC Patnaik D Watson LA Gao F Pan L Wang J. HDAC1 modulates OGG1-initiated oxidative DNA damage repair in the aging brain and Alzheimer's disease. Nat Commun. (2020) 11:2484. doi: 10.1038/s41467-020-16361-y 32424276 PMC7235043

[36] Tao CC Cheng KM Ma YL Hsu WL Chen YC Fuh JL. Galectin-3 promotes Abeta oligomerization and Abeta toxicity in a mouse model of Alzheimer's disease. Cell Death differentiation. (2020) 27:192–209. doi: 10.1038/s41418-019-0348-z 31127200 PMC7206130

[37] Dominguez-Andres J Dos Santos JC Bekkering S Mulder WJM van der Meer JWM Riksen NP. Trained immunity: adaptation within innate immune mechanisms. Physiol Rev. (2023) 103:313–46. doi: 10.1152/physrev.00031.2021 35981301

[38] Inoue Y Shue F Bu G Kanekiyo T. Pathophysiology and probable etiology of cerebral small vessel disease in vascular dementia and Alzheimer's disease. Mol neurodegeneration. (2023) 18:46. doi: 10.1186/s13024-023-00640-5 PMC10334598 37434208

[39] Zhang LY Pan J Mamtilahun M Zhu Y Wang L Venkatesh A. Microglia exacerbate white matter injury via complement C3/C3aR pathway after hypoperfusion. Theranostics. (2020) 10:74–90. doi: 10.7150/thno.35841 31903107 PMC6929610

[40] Iadecola C. The pathobiology of vascular dementia. Neuron. (2013) 80:844–66. doi: 10.1016/j.neuron.2013.10.008 PMC3842016 24267647

[41] Wang L Yang JW Lin LT Huang J Wang XR Su XT. Acupuncture attenuates inflammation in microglia of vascular dementia rats by inhibiting miR-93-mediated TLR4/myD88/NF-kappaB signaling pathway. Oxid Med Cell longevity. (2020) 2020:8253904. doi: 10.1155/2020/8253904 PMC7441436 32850002

[42] Washida K Hattori Y Ihara M. Animal models of chronic cerebral hypoperfusion: from mouse to primate. Int J Mol Sci. (2019) 20:6176. doi: 10.3390/ijms20246176 31817864 PMC6941004

[43] Qin C Fan WH Liu Q Shang K Murugan M Wu LJ. Fingolimod protects against ischemic white matter damage by modulating microglia toward M2 polarization via STAT3 pathway. Stroke. (2017) 48:3336–46. doi: 10.1161/STROKEAHA.117.018505 PMC5728178 29114096

[44] Shang J Yamashita T Fukui Y Song D Li X Zhai Y. Different associations of plasma biomarkers in alzheimer's disease, mild cognitive impairment, vascular dementia, and ischemic stroke. J Clin Neurol (Seoul Korea). (2018) 14:29–34. doi: 10.3988/jcn.2018.14.1.29 PMC5765253 29629537

[45] Vishnu VY Modi M Garg VK Mohanty M Goyal MK Lal V. Role of inflammatory and hemostatic biomarkers in Alzheimer's and vascular dementia - A pilot study from a tertiary center in Northern India. Asian J Psychiatr. (2017) 29:59–62. doi: 10.1016/j.ajp.2017.04.015 29061429

[46] Zhang Y Zhang H Zhao S Qi Z He Y Zhang X. S-nitrosylation of septin2 exacerbates aortic aneurysm and dissection by coupling the TIAM1-RAC1 axis in macrophages. Circulation. (2024) 149:1903–20. doi: 10.1161/CIRCULATIONAHA.123.066404 38357802

[47] Kurdi AT Bassil R Olah M Wu C Xiao S Taga M. Tiam1/Rac1 complex controls Il17a transcription and autoimmunity. Nat Commun. (2016) 7:13048. doi: 10.1038/ncomms13048 27725632 PMC5062600

[48] Wang T Yu H Hughes NW Liu B Kendirli A Klein K. Gene essentiality profiling reveals gene networks and synthetic lethal interactions with oncogenic ras. Cell. (2017) 168:890–903 e15. doi: 10.1016/j.cell.2017.01.013 28162770 PMC5445660

[49] Ciarlantini MS Barquero A Bayo J Wetzler D Dodes Traian MM Bucci HA. Development of an Improved Guanidine-Based Rac1 Inhibitor with *in vivo* Activity against Non-Small Cell Lung Cancer. ChemMedChem. (2021) 16:1011–21. doi: 10.1002/cmdc.202000763 33284505

[50] Bu F Munshi Y Furr JW Min JW Qi L Patrizz A. Activation of neuronal Ras-related C3 botulinum toxin substrate 1 (Rac1) improves post-stroke recovery and axonal plasticity in mice. J neurochemistry. (2021) 157:1366–76. doi: 10.1111/jnc.15195 PMC7982352 32964455

[51] Bu F Min JW Razzaque MA El Hamamy A Patrizz A Qi L. Activation of cerebral Ras-related C3 botulinum toxin substrate (Rac) 1 promotes post-ischemic stroke functional recovery in aged mice. Neural regeneration Res. (2024) 19:881–6. doi: 10.4103/1673-5374.382256 PMC10664129 37843224

[52] Zhou Y Castonguay P Sidhom EH Clark AR Dvela-Levitt M Kim S. A small-molecule inhibitor of TRPC5 ion channels suppresses progressive kidney disease in animal models. Science. (2017) 358:1332–6. doi: 10.1126/science.aal4178 PMC6014699 29217578

[53] Han W Ding P Xu M Wang L Rui M Shi S. Identification of eight genes encoding chemokine-like factor superfamily members 1-8 (CKLFSF1-8) by in silico cloning and experimental validation. Genomics. (2003) 81:609–17. doi: 10.1016/s0888-7543(03)00095-8 12782130

[54] Li L Hu Y Chen D Zhu J Bao W Xu X. CMTM5 inhibits the development of prostate cancer via the EGFR/PI3K/AKT signaling pathway. Mol Med Rep. (2022) 25:17. doi: 10.3892/mmr.2021.12533 34791506 PMC8628290

[55] Fan Y Zou HQ. CMTM5 influences Hippo/YAP axis to promote ferroptosis in glioma through regulating WWP2-mediated LATS2 ubiquitination. Kaohsiung J Med Sci. (2024) 40:890–902. doi: 10.1002/kjm2.12889 39166861 PMC11895632

