# Supplementary material for: Integrated bioinformatics analysis and biological experiments to identify key immune genes in vascular dementia
Source: Front Immunol. 2025 Mar 24;16:1560438. doi: 10.3389/fimmu.2025.1560438 (PMC11973090; doi:10.3389/fimmu.2025.1560438)
Supplement: Supplementary file 1 [file Table1.docx]

Raw data are available from the Jianguoyun platform:

https://www.jianguoyun.com/p/DZroBqwQ4quCChjIkegFIAA
